# The Effects of Piroxicam and Deracoxib on Canine Mammary Tumour Cell Line

**DOI:** 10.1100/2012/976740

**Published:** 2012-11-07

**Authors:** Fulya Üstün Alkan, Oya Üstüner, Tülay Bakırel, Suzan Çınar, Gaye Erten, Günnur Deniz

**Affiliations:** ^1^Department of Pharmacology and Toxicology, Faculty of Veterinary Medicine, Istanbul University, Avcılar, 34320 Istanbul, Turkey; ^2^Department of Immunology, Institute of Experimental Medicine, Istanbul University, Çapa, 34093 Istanbul, Turkey

## Abstract

Cyclooxygenase (COX) inhibitors, already widely used for the treatment of pain and inflammation, are considered as promising compounds for the prevention and treatment of neoplasia. The aim of our study was to determine the direct antiproliferative effects of nonsteroidal anti-inflammatory drugs (NSAIDs), piroxicam and deracoxib, at a variety of concentrations as both single and combined treatments on canine mammary carcinoma cell line CMT-U27 and to understand the mechanisms of cell death. MTT assay was performed to determine cell viability, and flow cytometric analyses were performed to evaluate apoptosis and cell cycle alterations. Significant decrease in cell viability was observed at high concentrations of piroxicam and deracoxib in both single and combined treatments after 72 h incubation. Combined treatment produced a significantly greater inhibition than that caused by either agent alone. Also apoptotic cell number was increased by both drugs at the cytotoxic concentrations. However, concomitant treatment of cells with piroxicam and deracoxib resulted in significant induction of apoptosis at lower concentrations and accumulation of cells in the G_0_/G_1_ phase. Significant cytotoxic effects exhibited by the combination of piroxicam and deracoxib against canine mammary carcinoma cells *in vitro* suggest an attractive approach for the treatment of canine mammary carcinoma.

## 1. Introduction 

 Canine mammary tumors are the most common malignant neoplasm constituting up to 52% of all tumors in female dogs [1]. Canine mammary carcinomas have epidemiologic, clinical, morphologic, and prognostic features similar to those of human breast cancer and therefore represent a comparative model to understand the underlying molecular mechanisms of carcinogenesis in both species [2]. Treatment for cancer of the mammary includes surgical excision, radiation therapy, and chemotherapy or a combination [3]. However, development of resistance to antitumour treatments has resulted in clinical failures, therefore; new treatments are needed for those animals who fail to respond to standard therapy or who initially present with metastatic disease [4]. 

Cyclooxygenase (COX) is an important enzyme that catalyzes the conversion of arachidonic acid to prostaglandin. Many studies have shown that a variety of tumors (breast, colon, lung, and bladder) produce greater amounts of prostaglandins than the normal tissue from which they were derived [5]. COX-1 is constitutively expressed in most tissues and regulates multiple physiologic processes, whereas COX-2 is induced by proinflammatory or mitogenic stimuli and overexpressed in a variety of cancers [6, 7]. In dogs, COX-2 is overexpressed in several mammary, rectal, bladder, cutaneous, and oral and ocular melanocytic tumors [8], and this expression has been associated with uncontrolled cell proliferation and differentiation, inhibition of apoptosis, increased angiogenesis and metastasis [9]. 

Nonsteroidal anti-inflammatory drugs (NSAIDs) that can block the activity of COXs are reported to have chemopreventive effects in several experimental studies and clinical trials [10–14]. Accordingly, the supression of COX-2 has been proposed to underlie the chemopreventive effects of NSAIDS. Recent reports suggest that the anticancer effects of NSAIDs and selective COX-2 inhibitors can occur through COX-independent pathways [15, 16]. However, evidence for the use of COX inhibitors in cancer prevention and the mechanism by which NSAIDs cause protective and anticarcinogenic effects are still to be determined.

Piroxicam is an NSAID used for treatment of osteoarthritis and also found to be effective in the treatment of transitional cell carcinoma of the bladder [17], in the treatment of inflammatory mammary carcinoma [18] and oral squamous cell carcinoma in dogs [19]. Also, it was shown to have antitumor activity against naturally acquired tumors in dogs in phase I and phase II clinical trials. In addition, high concentrations of piroxicam have been shown to inhibit cellular proliferation of canine osteosarcoma [20] and canine mammary carcinoma *in vitro *[21]. However, no significant reduction in cell growth occurred at concentrations that could be achieved *in vivo* without a significant risk of severe toxic side effects [21]. Deracoxib is a selective COX-2 inhibitor licensed for the treatment of pain and inflammation associated with osteoarthritis and orthopedic surgery in canines [22]. Deracoxib was shown to have *in vivo* antitumor activity and *in vitro* cytotoxic properties. Also, deracoxib was found to reduce the growth of canine mammary cancer xenografts in mice [14] and was found to be cytotoxic in osteosarcoma cell lines [20]. The present study, therefore, was designed to determine the antineoplastic mechanism of piroxicam and deracoxib especially to determine the efficiency of the combination of these drugs on canine mammary carcinoma cells. 

## 2. Materials and Methods 

### 2.1. Cell Line

The canine mammary carcinoma cell line CMT-U27 (a generous gift from Assoc Professor Eva Hellmén) was obtained from the Uppsala University, Sweden. CMT-U27 cell line was derived from a primary tumor (infiltrating ductal carcinoma) and when inoculated in the fat mammary pad of female *nude* mice, it metastasized to the lymph nodes, lungs, liver, and heart [23].

### 2.2. Cell Culture and Treatment

Mammary carcinoma cells, at passage 134, were cultured in Dulbecco's modified Eagle's medium-F12 (Sigma Chemicals, St. Louis, USA), supplemented with 2 mM L-glutamine (Sigma, St. Louis, USA), 10% fetal bovine serum (Biological Industries, Israel), 100 IU mL^−1^ penicillin G, 100 *μ*g mL^−1^ streptomycin, and 2.5 *μ*g mL^−1^ amphotericin B (Sigma, St. Louis, USA), and maintained in monolayer culture at 37°C in a humidified 5% CO_2_ atmosphere. Culture media was changed every 2-3 days for maintaining the exponential growth of the cells, and cells were subcultured as they reached 80–90% confluence. Subconfluent cells were passaged with 0.25% trypsin and 0.02% EDTA solution (Sigma, St. Louis, USA). Nonspecific COX inhibitor piroxicam (Sigma, St. Louis, USA) and selective COX-2 inhibitor deracoxib (Novartis, Pharmaceuticals Inc.) were dissolved in sterile dimethylsulfoxide (DMSO) to create a stock solution, filtered through 0.2 *μ*m filter, and stored at −20°C. The stock solution was diluted with the medium, and the cells were treated with 50, 100, 250, 500, and 1000 *μ*M concentrations of each compound for 72 h. Control group was cultured without piroxicam and deracoxib, and corresponding amount of DMSO was added to the medium. 

### 2.3. Cell Viability Assay

Cells at passage 138 were cultured at a density of 1 × 10^4^ cells/100 *μ*L in 96-well flat-bottom microtiter plates (Jet Biofil, Canada) and allowed to attach for 24 h. Thereafter, medium was removed and replaced with 100 *μ*L of medium containing 50, 100, 250, 500, and 1000 *μ*M concentrations of piroxicam and deracoxib in triplicate wells. After 72 h incubation, cell viability was assessed using cell proliferation kit (MTT, Roche, Germany), according to the manufacturer's instructions. 3-(4,5-dimethylthiazol-2-yl)-2,5-diphenyl-tetrazoliumbromide (MTT) test is based on the enzymatic reduction of the tetrazolium salt MTT to a formazan (1-[4,5-Dimethylthiazol-2-yl]-3,5-diphenylformazan) by mitochondria of living cells [24]. Briefly, 10 *μ*L of MTT solution 5 mg/mL in phosphate buffered saline (PBS) was added to each well and incubated for 4 h at 37°C in CO_2_ incubator. The purple water insoluble formazan salt was then dissolved with 10% SDS in 0.01 M HCl and incubated overnight in a humidified 5% CO_2_ atmosphere. The optical densities (OD) of the wells were measured at 550 nm by microplate reader (ELx800, Biotek Instruments, USA). The effect of each compound on growth inhibition was assessed as percent cell viability where vehicle-treated cells were taken as 100% viable. The dose-response curves were plotted for each drug, and the concentration of drug required for 50% inhibition of cell viability (IC_50_) was determined graphically. 

### 2.4. Apoptosis Assay

Flow cytometric analyses of phosphatidylserine exposure were quantitatively detected using Annexin V-fluorescein isothiocyanate (FITC) Apoptosis Detection Kit (BD Bioscience, San Jose, CA). The method is based on the binding of Annexin V to phosphatidylserine that is translocated from the inner membrane leaflet to the outer layer in cells undergoing apoptosis [25]. The cells were cultured at a density of 1 × 10^5^/mL in 24-well flat-bottom microtiter plates (Jet Biofil, Canada) and cultivated in medium as described above. After 24 h, medium was replaced with fresh medium containing 50–1000 *μ*M concentrations of piroxicam and deracoxib. The cells were trypsinized 72 h after the treatment, washed twice each with ice cold PBS, and then resuspended in binding buffer (0.1 M Hepes/NaOH (pH 7.4), 1.4 M NaCl, 25 mM CaCl_2_), supplemented with 5 *μ*L of FITC-Annexin V and 5 *μ*L of propidium iodide (PI). The cell suspension was gently vortexed and incubated for 15 min at room temperature in the dark. Following the incubation, 400 *μ*L of binding buffer was added to each tube and then analyzed within 1 h on a FACScan flow cytometer (BD Biosciences) using the standard optics for detecting FL1 (FITC) and FL2 (PI). Data were analyzed with CellQuest WinMDI software (BD Biosciences, San Jose, CA). 

### 2.5. Cell Cycle Analyses

Cell cycle alterations were detected using Coulter DNA Prep Reagents Kit (Beckman Coulter, UK) by flow cytometry. The cells were cultured at a density of 1 × 10^5^/mL in 24-well flat-bottom microtiter plates (Jet Biofil, Canada) and cultivated and treated in medium as described in apoptosis assay. After the 72 h treatment, the floating and adherent cells were combined for the analyses. Cells were washed with PBS, and the cell suspensions were resuspended in 100 *μ*L of PBS. The resuspended cells were stained according to the manufacturer's instructions. DNA-prep LPR (Lyse) (100 *μ*L) was added to the tube, the cell suspension was vortexed, and then 1 mL DNA-prep stain (propidium iodide + RNase) (1 mL) was added. The cells incubated for 30 min at room temperature in the dark prior to linear data acquisition on a FACScan flow cytometer (BD Biosciences). A minimum of 10.000 events were acquired for each sample [6]. The distribution of cells in the different cell-cycle phases was analyzed from the DNA histograms using CellQuest WinMDI software (BD Biosciences, San Jose, CA). 

### 2.6. Statistical Analyses

Samples were assayed at least three times for each determination, and results were expressed as the mean ± SEM. The statistical differences between the treatments and the control were tested by one-way analysis of variance (ANOVA) followed by the Student's *t*-test using the “Instat” statistical computer program. A difference in the mean *P*-values of 0.05 or less was considered to be statistically significant.

## 3. Results 

### 3.1. Cell Viability Assay

**Figure 1:**
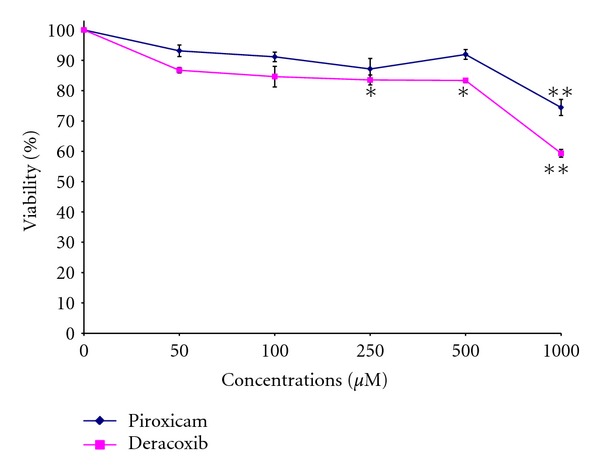
The effects of piroxicam and deracoxib on the proliferation of the canine mammary carcinoma cell line CMT-U27. Data are expressed as the percentage of inhibition compared with control in which cell survival was assumed as 100%. *P < 0.05, **P < 0.01 (*n* = 3).

The effects of piroxicam and deracoxib on cell proliferation rates of CMT-U27 canine mammary carcinoma cells were shown in Figure 1. After 72 h incubation, significant reductions were seen at 250, 500, and 1000 *μ*M doses of deracoxib by 16.49%, 16.64%, and 40.69% of the control level, respectively, whereas 1000 *μ*M concentration of piroxicam decreased the cell viability 25.55%. 

**Figure 2:**
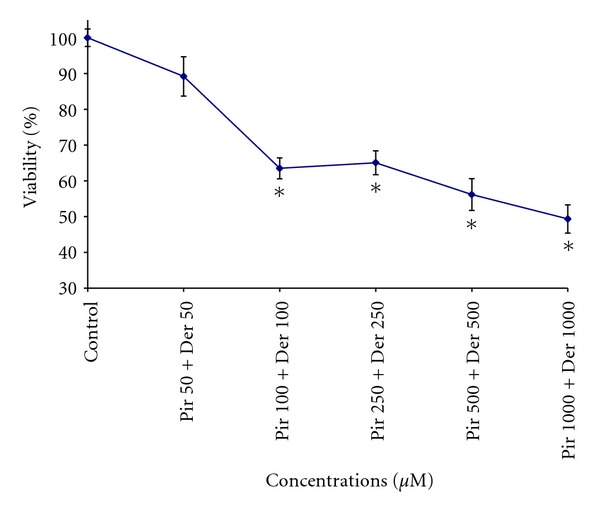
Antiproliferative effects of coadministration of piroxicam (Pir) and deracoxib (Der) on canine mammary carcinoma cell line after 72 h incubation. Data are expressed as the percentage of inhibition compared with control in which cell survival was assumed as 100%. *P < 0.05 (*n* = 3).

IC_50_ value for deracoxib was found as 974.481 *μ*M. However, as a maximal inhibitory effect of piroxicam was not observed in the concentration range studied, the corresponding IC_50_ value could not be calculated. When the 2 inhibitors were combined, a significant decrease in cell growth was observed at 100, 250, 500, and 1000 *μ*M concentrations (36.5%, 34.94%, 43.84%, and 50.66%, resp.) (Figure 2). The combination of both COX inhibitors exhibited a synergistic effect in CMT-U27 cells. 

### 3.2. Apoptosis Assay

**Figure 3:**
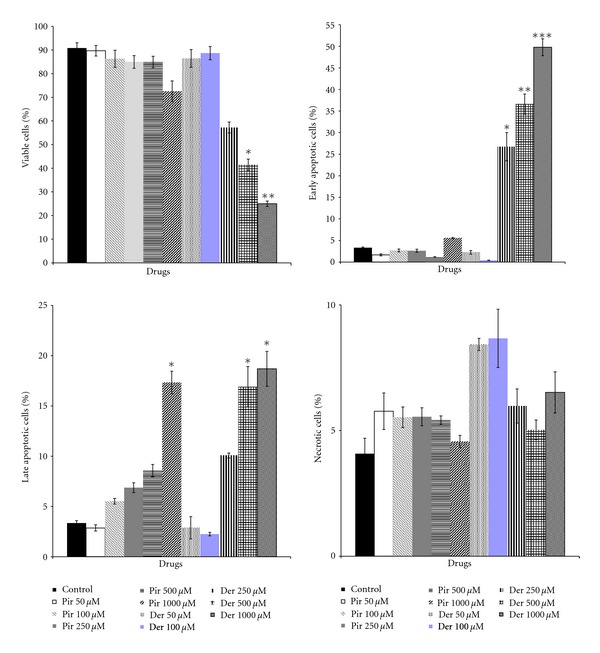
Flow cytometric analysis of apoptosis of CMT-U27 cells after treatment with piroxicam (Pir) and deracoxib (Der) for 72 h. Data are expressed as mean values ± standard error of means shown with *P < 0.05, **P < 0.01,   and  ***P < 0.001 versus control group (*n* = 3).

The apoptotic cell number of CMT-U27 cells after 72 h incubation in the presence or absence of piroxicam and deracoxib at various concentrations (50–1000 *μ*M) was shown in Figure 3. Piroxicam at 1000 *μ*M concentration (*P* < 0.05), deracoxib at 250 *μ*M, and higher concentrations (*P* < 0.01, *P* < 0.001) decreased the number of viable cells and increased the number of apoptotic cells as a sum of early and late apoptotic cells significantly.

**Figure 4:**
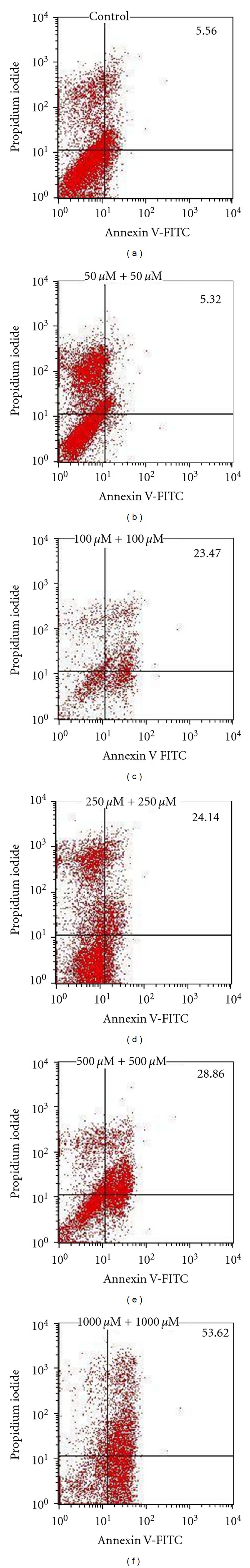
Flow cytometric analysis of apoptosis of coadministration of piroxicam and deracoxib on canine mammary carcinoma cell line after 72 h incubation. The lower left quadrant of the histogram shows the viable cells, which exclude PI, and are negative for FITC-Annexin-V-binding FITC^−^/PI^−^ (LL). The lower right compartment represents the early apoptotic cells, which are PI negative and Annexin-V positive, indicating the translocation of phosphatidylserine to the external cell surface FITC^+^/PI^−^ (LR). The upper right quadrant represents the late-stage apoptotic cells which are PI and Annexin-V positive FITC^+^/PI^+^ (UR), and the upper left compartment shows the necrotic cells which are only PI positive FITC^−^/PI^+^ (UL). The numbers written on histograms represent the sum of early and late apoptotic cells (%).

Whereas, again, the combination of piroxicam and deracoxib (100 *μ*M and higher) exhibited a significant increase in the apoptotic activity. The percentages of apoptotic cells were 5.56% (control), and 5.32% (piroxicam 50 *μ*M + deracoxib 50 *μ*M), 23.47% (piroxicam 100 *μ*M + deracoxib 100 *μ*M), 24.14% (piroxicam 250 *μ*M + deracoxib 250 *μ*M), 28.86% (piroxicam 500 *μ*M + deracoxib 500 *μ*M), 53.62% (piroxicam 1000 *μ*M + deracoxib 1000 *μ*M).  Figure 4 shows representative results of flow cytometry for the apoptosis of CMT-U27 cells after incubation in the presence of piroxicam and deracoxib.

### 3.3. Cell Cycle Analyses

Cell cycle phase distribution was evaluated to assess which cell cycle changes contribute to CMT-U27 cell number reduction by piroxicam and deracoxib, and cellular DNA contents were examined by flow cytometry. The results showed that treating cells with piroxicam and deracoxib at 500 and 1000 *μ*M concentrations caused a significant inhibition of cell cycle progression in CMT-U27 cells at 72 h (Table 1) resulting in a clear increase of the percentage of cells in the G_0_/G_1_ phase when compared with the control. The cell cycle evaluation in CMT-U27 cell line showed an average: 45.23%, 35.62%, and 19.16% of cells in G_0_/G_1_, S, and G_2_/M phases, respectively. 

Treatment with the combination of 1000 *μ*M concentrations of piroxicam and deracoxib resulted in an increase in the number of cells in the G_0_/G_1_ phase and a decrease in the number of cells in the S and G_2_/M phase indicating the cell cycle arrest at G_0_/G_1_ phase correlating with the induction of apoptosis (Table 2). At the highest dose combination of two drugs, the percentage of cells in the G_0_/G_1_ phase was increased from 63.8 to 89.95% when compared with control. Conversely, the percentage of cells in S phase decreased from 22.48% to 8.27%, and G_2_/M phase cells decreased from 13.72% to 1.78%. 

## 4. Discussion 

In recent years, experimental, epidemiological, and clinical studies have identified COX inhibitors as promising compounds in anticancer therapy. There is an ample evidence of the involvement of COX-1 and COX-2 in carcinogenesis for many different types of malignant tumor, and results of numerous studies indicate that various NSAIDS exert antiproliferative and antineoplastic effects on several canine cancer cell lines [20, 21, 26]. These findings have raised the possibility that COX inhibitors might also act as tumor suppressors. Deracoxib, a selective COX-2 inhibitor, and piroxicam, a nonselective COX inhibitor, and also frequently used drugs in veterinary medicine were evaluated to determine the cytotoxic effects on canine mammary carcinoma cells. 

In the present study, we investigated firstly the *in vitro* effects of piroxicam and deracoxib on canine mammary carcinoma cell line CMT-U27. As a result, both drugs suppressed proliferation of canine mammary tumor cells in a concentration-dependent manner. No significant difference in cell viability was seen after incubating cells with piroxicam (50–500 *μ*M) and deracoxib (50–100 *μ*M), but highly significant cell proliferation inhibition was seen after 72 h incubation with 1000 *μ*M piroxicam and 250, 500, and 1000 *μ*M deracoxib. In a previous investigation, it was shown that piroxicam inhibited cellular proliferation of canine mammary carcinoma *in vitro *at concentrations above 1 *μ*g/mL and induced apoptosis in a dose-dependent manner, and the authors suggested that the significant inhibition of proliferation and induction of apoptosis could only occur when drug concentrations were in excess that can be achieved *in vivo* following maximum recommended dose rate of piroxicam [21]. Royals and colleagues [20] reported that piroxicam and deracoxib significantly decreased the cell proliferation of highly metastatic canine osteosarcoma cells at ≥500 *μ*M and ≥100 *μ*M concentrations, respectively. This effect was similar to the effect reported by Wolfesberger and colleagues [27] that high concentrations of meloxicam (400 and 600 *μ*M) showed a clear antiproliferative and pro-apoptotic effect on canine osteosarcoma cells *in vitro*. Also a high IC_50_ (615 *μ*M) was reported for piroxicam against canine squamous cell carcinoma cell line [26], and although this concentration can never be achieved in the serum of dogs, reduction on tumour volumes was seen in a xenograft model of canine mammary tumours in piroxicam-treated mice [14]. In contrast to these findings, deracoxib at a dose of 6 mg/kg had no effect on the growth of canine mammary tumour xenografts in nude mice [14]. Our findings correspond to previous studies that highly significant inhibition of cell proliferation was obtained only at higher concentrations of piroxicam and deracoxib. The observed cytotoxic effects of piroxicam and deracoxib at various concentrations can be cell-type specific as demonstrated by the variation in responses by different tumour cell lines reported in several studies [20, 21]. 

The mechanism responsible for piroxicam and deracoxib-mediated cell proliferation inhibition in the present study is not known. To elucidate the growth inhibition of CMT-U27 cells by piroxicam and deracoxib, apoptosis assay and cell cycle analyses were performed. Increases in the number of apoptotic cells reached significance (*P* < 0.05) at doses of piroxicam of 1000 *μ*M and at doses of deracoxib 250 *μ*M above for CMT-U27 cells. However, concentrations required for induction of apoptosis as single treatments of both drugs were high. In combination experiments, the apoptotic effect was seen at much lower concentrations as above 100 *μ*M than that seen with piroxicam and deracoxib as single treatment. The increase in both early and late apoptotic activity seen in CMT-U27 cells with combination treatment indicated that regulation in apoptosis is at least partially responsible for their effect. An increase in early apoptotic activity indicates that the cells are in a static, nonproliferative state, while increases in late apoptotic activity suggest that the cells are in the final stages of the apoptotic, cycle and the cell death is imminent [28]. Experimental studies indicated that apoptosis induction is one of the proposed effects of NSAIDS used in chemoprevention studies [29, 30]. Several mechanisms are described as playing a role in the induction of apoptosis and the mitochondrial pathway seems to be the most common apoptotic mechanism. Cytotoxic stress leads to expression of the proteins of the Bcl family that acts independently or in complexes on the mitochondrial membrane. The degree of apoptosis was shown to correlate with the levels of the expression of Bcl-2, main antiapoptotic protein, and its overexpression in cancer cells ensures their resistance to chemotherapy [31]. As reported in a previous study, Bcl-2 expression was high in CMT-U27 cells [32], thus correlating with the results of our study that high doses of piroxicam and deracoxib were required for the induction of apoptosis in response to apoptotic stimulus. The positive association between apoptosis and proliferation suggests that apoptosis might reflect not only cell loss but also the proliferative activity. 

More recent data with non-COX inhibiting NSAIDs and the effectiveness of NSAIDs in COX-deficient cell lines indicated that NSAID-induced growth inhibition and apoptosis may be occurring through COX-independent mechanisms such as cell cycle arrest and inhibition of angiogenesis [33, 34]. In our study, although piroxicam inhibited the cell proliferation dose-dependently, the inhibition was associated with cell death only at the higher dose, suggesting that the drug may inhibit the cell growth by retarding cell cycle progression. Our flow cytometric analyses showed that both drugs at 500 and 1000 *μ*M induced G_0_/G_1_ arrest with no significant effect on G_2_/M transition. Conversely, the percentage of cells in S phase were significantly decreased. However, when these two agents were combined, greater effects were seen. A significant increase of cells in the G_0_/G_1_ phase and a significant decrease of cells entering the S phase and G_2_/M phase were observed showing that the cells were at rest. The cell cycle alterations were achieved at both drug concentrations that repressed CMT-U27 cells growth, indicating that cell cycle arrest is one of the primary mechanisms responsible for the antiproliferative action of deracoxib and piroxicam. Krol et al. [32] reported that growth rate (short cell cycle) and antiapoptotic potential of CMT-U27 cell line were high and spontaneous and induced apoptosis was low. The authors observed that the high growth rate and antiapoptotic potential in CMT-U27 cells were associated with enhanced expression of genes-involved Ca^2+^ signaling pathway (Calmodulin 1, 2, 3, and SPSB2) and growth hormone cellular pathway. The cell cycle length of CMT-U27 line was reported as 53.4 hour, and the distribution of cells was G_0_/G_1_ 64%, S phase 15%, and G_2_/M 20%. In the present study, similar to those reported results we found the percentage of cells 63.8%, 22.48%, and 13.72% in G_0_/G_1_, S, and G_2_/M phase, respectively. 

Although antiproliferative and apoptotic effects were seen with both drugs, the concentrations we used to obtain significant effects appear to be too high to be achieved *in vivo*, therefore our results cannot be directly extrapolated to dogs but can provide insight into potential mechanisms of NSAID action in mammary cancer cells. However, intralesional or topical therapy may be appropriate in some types of tumours, and local administration of the drug could increase the concentration to which the tumour is exposed and minimise side effects. 

In the present study, the combination of the two inhibitors significantly increased the response over that observed with single agents, suggesting that combined treatment with COX inhibitors may be an attractive approach for the treatment of canine mammary carcinoma. Further, *in vivo* clinical studies should be considered before recommending their clinical use either alone or in combination with other agents for treatment of mammary cancer in dogs.

## Figures and Tables

**Table 1:** Effects of piroxicam and deracoxib on cell cycle phase distribution of CMT-U27 cells. Data are expressed as mean values ± standard error of means shown with *P < 0.05 versus control group.

Drug concentrations	% G_0_/G_1_	% S	% G_2_/M
Control	45.23 ± 1.17	35.62 ± 2.57	19.16 ± 1.81
Piroxicam (50 *μ*M)	55.93 ± 2.79	35.49 ± 1.92	8.58 ± 0.83
Piroxicam (100 *μ*M)	58.44 ± 2.48	34.63 ± 2.25	6.93 ± 1.03
Piroxicam (250 *μ*M)	70.31 ± 1.32	24.13 ± 2.06	5.56 ± 0.39
Piroxicam (500 *μ*M)	80.22 ± 1.67*	8.46 ± 0.77*	11.32 ± 1.78
Piroxicam (1000 *μ*M)	79.05 ± 2.19*	11.93 ± 0.84*	9.02 ± 0.51
Deracoxib (50 *μ*M)	50.21 ± 2.55	41.35 ± 1.92	8.44 ± 1.10
Deracoxib (100 *μ*M)	53.74 ± 2.12	42.51 ± 1.77	3.75 ± 0.29
Deracoxib (250 *μ*M)	65.57 ± 1.45	15.04 ± 1.55	19.39 ± 1.29
Deracoxib (500 *μ*M)	79.94 ± 2.14*	14.03 ± 2.37	6.03 ± 0.53
Deracoxib (1000 *μ*M)	71.34 ± 2.36*	4.72 ± 0.85*	23.94 ± 2.26

**Table 2:** Effects of piroxicam and deracoxib combination on cell cycle phase distribution of CMT-U27 cells. Data are expressed as mean values ± standard error of means shown with *P < 0.05 versus control group.

Drug concentrations	% G_0_/G_1_	% S	% G_2_/M
Control	63.8 ± 2.11	22.48 ± 1.52	13.72 ± 1.50
Piroxicam + deracoxib (50 + 50 *μ*M)	67.4 ± 3.04	19.97 ± 1.63	12.63 ± 1.26
Piroxicam + deracoxib (100 + 100 *μ*M)	64.53 ± 2.51	22.27 ± 1.22	13.2 ± 1.10
Piroxicam + deracoxib (250 + 250 *μ*M)	69.58 ± 3.50	17.75 ± 1.45	12.67 ± 0.76
Piroxicam + deracoxib (500 + 500 *μ*M)	72.2 ± 2.17	19.95 ± 1.12	7.85 ± 1.51
Piroxicam + deracoxib (1000 + 1000 *μ*M)	89.95 ± 3.35*	8.27 ± 1.81*	1.78 ± 0.68*
